# Astrocytic HSP90AA1 Upregulation and Altered Synaptic Signaling in Parkinson’s Disease: Transcriptomic Screening and In Vivo Validation

**DOI:** 10.3390/ijms27167140

**Published:** 2026-08-09

**Authors:** Yiyuan Xu, Yanfeng Shi, Yan Li, Jia Luo, Wei-Na Jin

**Affiliations:** 1 Center for Neurological Diseases, China National Clinical Research Center for Neurological Diseases, Beijing Tiantan Hospital, Capital Medical University, Beijing 100070, China; 2 Changsha Blood Center, Changsha 410024, China

**Keywords:** Parkinson’s disease (PD), WGCNA, bioinformatics, single-cell sequencing analysis, synapse

## Abstract

Parkinson’s disease (PD) is a multisystem disorder in which gastrointestinal dysfunction often precedes motor symptoms, yet the molecular links between peripheral stress and central neurodegeneration remain unclear. We investigated whether genes commonly dysregulated in PD and a classic model of intestinal inflammation (IBD) might reveal conserved stress-responsive molecules relevant to brain pathology. Shared gene signatures between PD and inflammatory bowel disease (IBD) were identified from peripheral blood transcriptomes using weighted gene co-expression network analysis (WGCNA). Hub genes were prioritized via protein–protein interaction (PPI) analysis and evaluated for expression consistency in independent brain tissue transcriptomic datasets. Single-cell RNA sequencing (scRNA-seq) of the PD substantia nigra was used to define the cellular context of the key hub gene, and CellChat analysis assessed intercellular communication changes. Immunofluorescence validation was performed in an MPTP-induced PD mouse model. We identified 79 shared genes and 6 hub genes, among which only HSP90AA1 showed consistent upregulation across independent PD transcriptomic validation datasets. Functional enrichment highlighted inflammation-related pathways. Because peripheral immune infiltration showed only minor changes, we further investigated the cellular context of HSP90AA1 within the PD brain. ScRNA-seq analysis of the PD substantia nigra demonstrated that HSP90AA1 was expressed across multiple cell populations. Integration with transcriptional regulatory analysis identified TP53 as a potential upstream regulator, and the strongest TP53–HSP90AA1 co-expression and cellular colocalization signals were observed in astrocytes, prompting further astrocyte-focused investigation. CellChat analysis revealed altered intercellular communication patterns in PD substantia nigra, including changes in synapse-associated ligand–receptor interaction signatures, particularly involving NCAM-related pathways. In the MPTP-induced PD mouse model, immunofluorescence identified astrocytic HSP90α upregulation, and increased nuclear p53 signal in astrocytes, accompanied by dopaminergic neuron loss. Conclusion: Astrocytic upregulation of HSP90AA1 is associated with altered synapse-related intercellular communication patterns in the PD substantia nigra, potentially involving a predicted TP53 associated regulatory component. These findings, validated in an MPTP mouse model, identify HSP90AA1 as a candidate stress-responsive hub linking peripheral inflammatory states with astrocyte-associated molecular alterations in PD, providing a framework for further experimental investigation.

## 1. Introduction

Parkinson’s disease (PD) is a prevalent neurodegenerative disorder characterized by hallmark motor symptoms such as tremor and bradykinesia. As one of the fastest-growing neurological conditions globally, its pooled all-age prevalence reaches approximately 1.51‰ worldwide, with over 11.7 million affected individuals in 2021 and mounting disability burden across all continents [1,2]. However, there is still no available disease-modifying treatment to halt or reverse its progression. The pathological hallmarks of PD include the selective degeneration of dopaminergic neurons in the substantia nigra pars compacta (SNpc) and the formation of Lewy bodies, which are intracellular inclusions primarily composed of misfolded α-synuclein [3,4]. In addition to the classic motor symptoms, a range of non-motor symptoms, including gastrointestinal dysfunction, frequently occur throughout the disease course and may precede motor manifestations in a subset of patients [5,6,7,8].

Emerging evidence suggests that chronic intestinal inflammation and dysbiosis contribute to PD pathogenesis [9,10]. Inflammatory bowel disease (IBD), a classic model of chronic relapsing intestinal inflammation, is epidemiologically linked to an increased risk of PD [11,12,13]. Moreover, a plethora of experimental evidence points to α-synuclein deposition in the gastrointestinal system [14,15,16,17,18]. The gut–brain axis hypothesis provides a framework describing potential bidirectional interactions among gastrointestinal alterations, immune responses, and central nervous system (CNS) pathology [19,20,21]. Given these observations, we hypothesized that genes co-dysregulated in PD and IBD may represent shared stress-responsive molecular signatures associated with inflammatory stress. Characterizing such shared signals in the CNS could provide insight into PD-relevant brain alterations without implying direct gut-to-brain transmission.

In this study, we integrated peripheral blood transcriptomic datasets from PD and IBD to identify shared gene signatures. Hub genes were prioritized through protein–protein interaction analysis and validated in independent PD cohorts. ScRNA-seq of the PD SNpc was subsequently performed to define the cellular context of the key hub gene, and CellChat analysis was used to infer potential alterations in cell–cell communication patterns. The TRRUST database was utilized to predict upstream transcription factors (TFs) [22]. An MPTP-induced PD mouse model was employed for immunofluorescence validation.

We identified HSP90AA1 as the only hub gene consistently upregulated in independent PD validation cohorts. In the PD SNpc, HSP90AA1 expression showed astrocyte-associated enrichment and was linked to altered synapse-related communication patterns. Bioinformatic analysis predicted a TP53-mediated regulatory component in astrocytes, and immunofluorescence in MPTP-treated mice identified astrocytic HSP90α upregulation alongside dopaminergic neuron loss. These findings identify astrocyte-associated HSP90AA1 as a candidate stress-responsive molecule potentially involved in PD-related neuronal communication alterations, providing a framework for future mechanistic investigation.

## 2. Results

### 2.1. Identification of Shared Co-Expressed Genes and Hub Genes in PD and IBD

**Figure 1:**
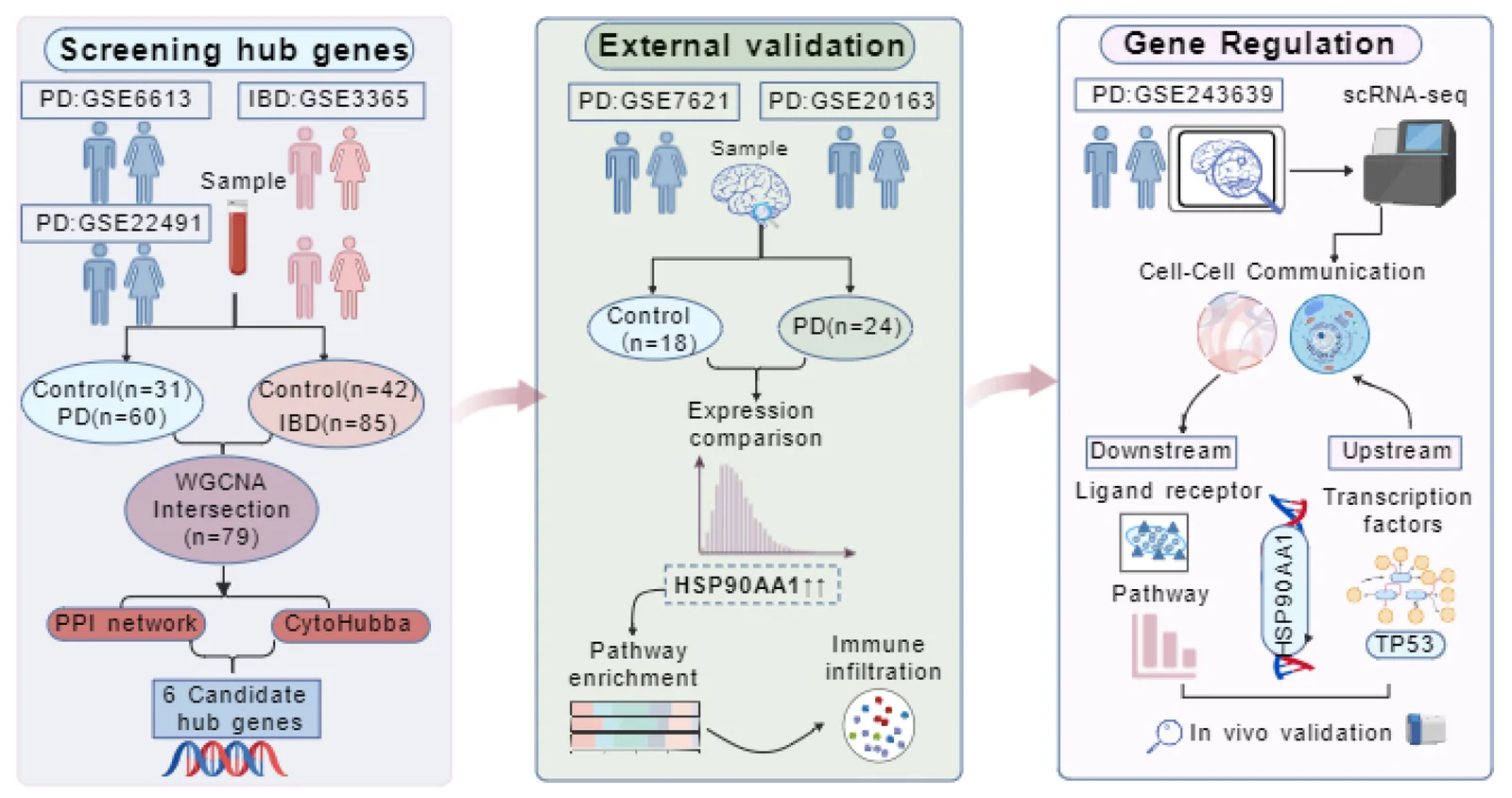
Flowchart of the study design. PD: Parkinson’s disease, IBD: Inflammatory bowel disease, WGCNA, weighted gene co-expression network analysis, PPI, protein–protein interaction.

**Figure 2:**
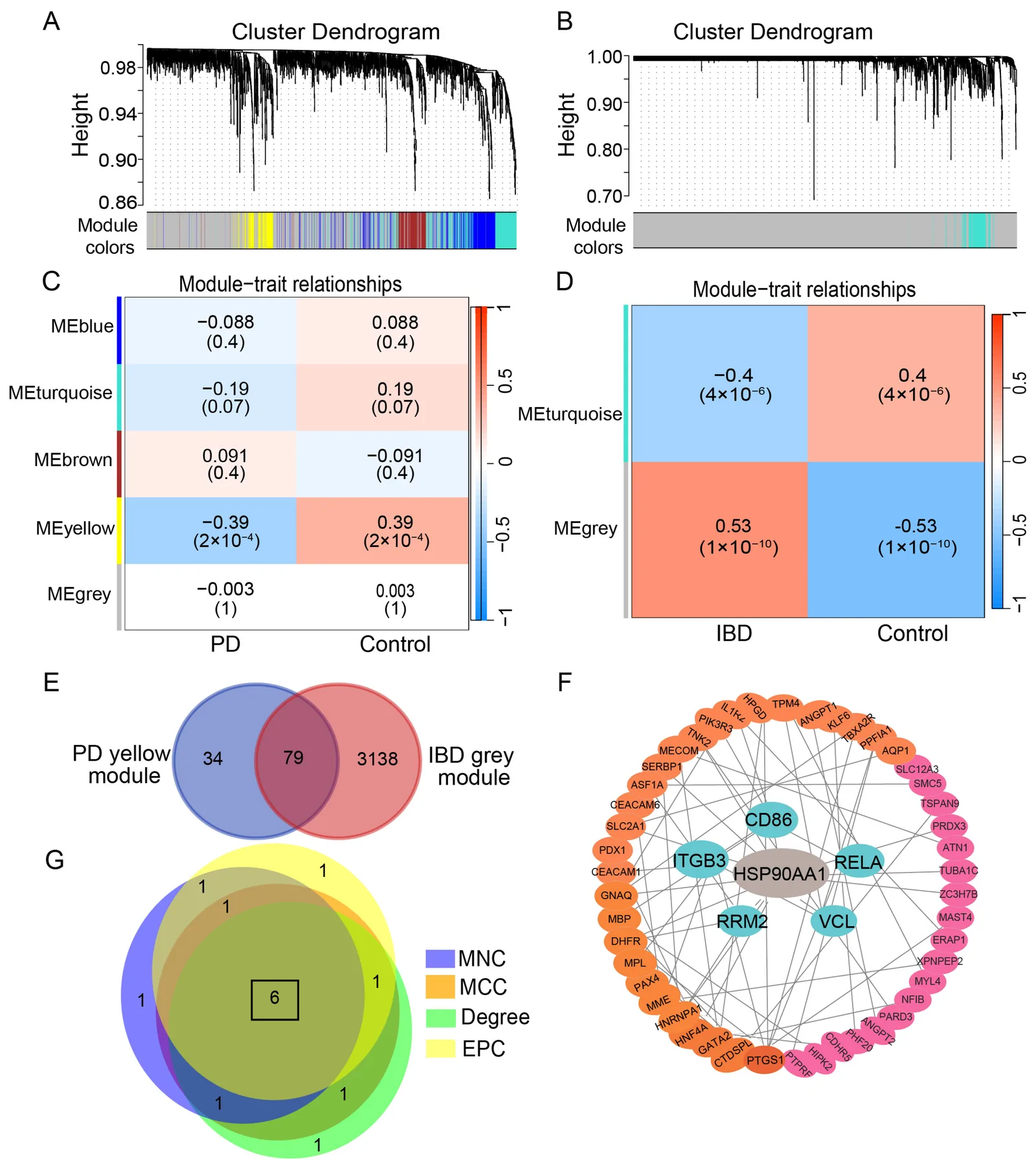
Co-expression module analysis and core gene screening in PD and IBD. (**A**,**B**) Cluster dendrogram of co-expressed genes and module assignment (colors below the dendrogram indicate initial modules before merging) for PD (**A**) and IBD (**B**). (**C**,**D**) Module-trait relationship heatmaps showing Pearson correlations between module eigengenes and disease status (PD vs. control). Color intensity indicates Pearson correlation strength (red = positive, blue = negative; color bar shows coefficient range). Numerical values within each cell represent correlation coefficients; values given in parentheses are corresponding statistical *p*-values. (**E**) Venn diagram displaying overlapping and unique genes from PD yellow module and IBD grey module; numbers within each segment denote gene counts, with 79 overlapping shared genes in the intersection region. (**F**) Protein–protein interaction (PPI) network of the 79 overlapping genes. Constructed via STRING. Nodes represent individual genes; connecting lines indicate predicted protein-protein interactions. (**G**) Venn diagram for hub-gene screening using four CytoHubba algorithms (Degree, MCC, MNC, EPC). Numbers in each region indicate gene counts yielded by each algorithm; the boxed number in the center represents six candidate hub-genes shared across all four algorithms.

The study workflow is illustrated in Figure 1. After data preprocessing (batch effect correction via ComBat) and outlier removal (1 sample excluded from PD datasets; Supplementary Figure S1A), the final cohort included 60 PD patients, 31 PD controls, 85 IBD patients, and 42 IBD controls (Table 1). WGCNA identified disease-associated modules in PD and IBD, and intersection of these modules yielded 79 shared genes dysregulated in both diseases (Figure 2A–D). These genes were visualized in a PPI network (STRING; Figure 2F). Using four centrality algorithms (MCC, MNC, EPC, and Degree), six hub genes were identified: HSP90AA1, ITGB3, RRM2, CD86, RELA, and VCL (Figure 2F).

### 2.2. Validation of Hub Genes

**Figure 3:**
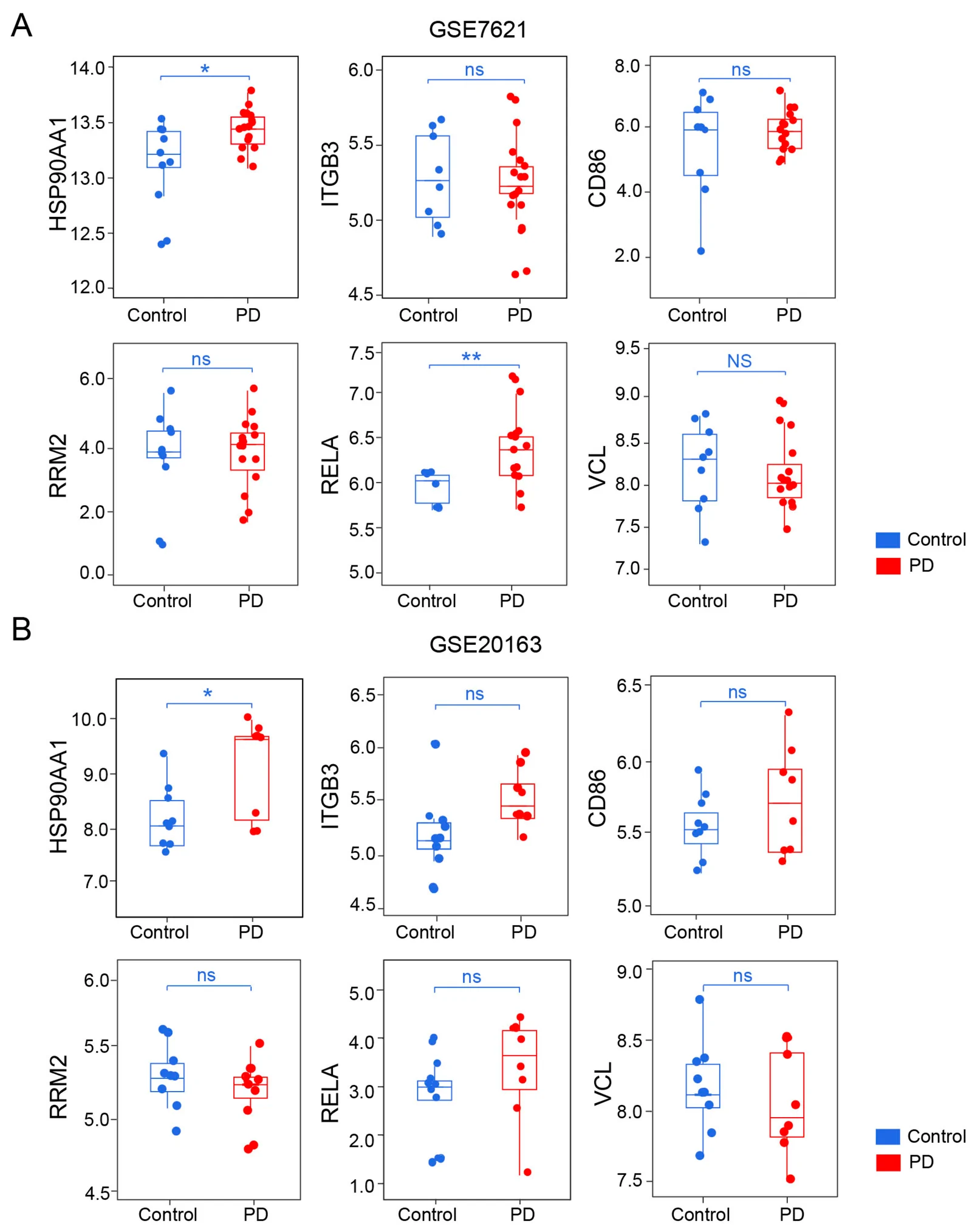
Expression patterns of WGCNA-derived hub genes in two independent PD brain transcriptomic validation datasets. (**A**): GSE7621; (**B**): GSE20163). Box plots with overlayed individual sample points show normalized expression of each gene in control (blue) and PD (red) groups. Statistical comparisons between two groups were performed using the two-sided Wilcoxon rank-sum test. * *p* < 0.05, ** *p* < 0.01, NS = non-significant.

Independent PD validation cohorts demonstrated that only HSP90AA1 showed consistent upregulation among the six hub genes (*p* < 0.05; Figure 3A,B). Specifically, HSP90AA1 expression was significantly increased in the substantia nigra of PD patients in both GSE7621 (16 PD vs. 9 controls) and GSE20163 (8 PD vs. 9 controls). No consistent expression differences were observed for the other five hub genes, supporting HSP90AA1 as the primary candidate for downstream analyses.

### 2.3. Functional Enrichment and Preliminary Immune Profiling

GO/KEGG enrichment analysis of the 79 shared genes highlighted biological processes related to inflammation and tissue repair (e.g., wound healing, hemostasis; Supplementary Figure S2A) and pathways including Rap1 and HIF-1 signaling (key inflammation-related pathways; Supplementary Figure S2B).

**Figure 4:**
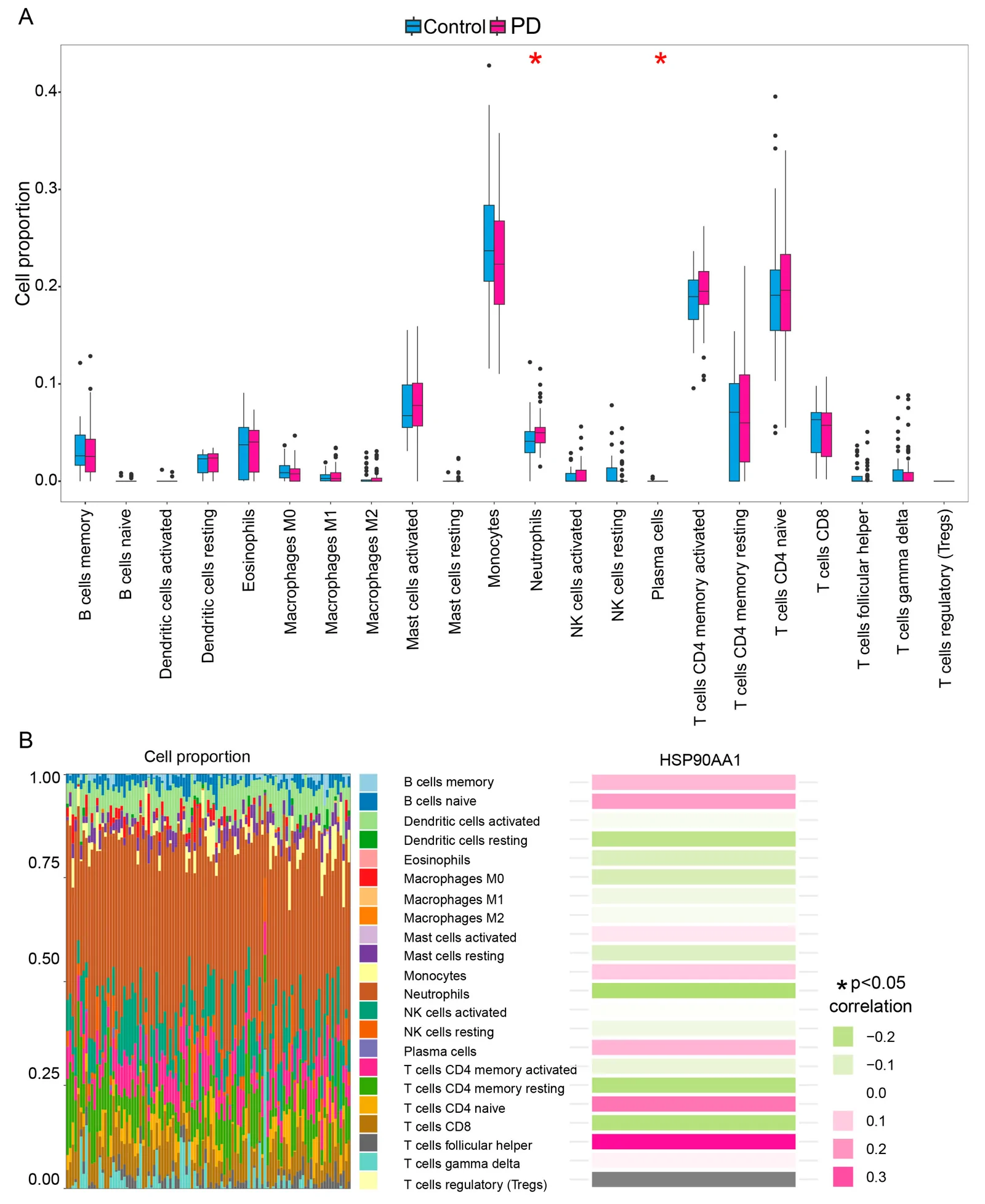
Immune cell infiltration analysis in PD datasets via CIBERSORT. (**A**) Box plots showing the proportions of 22 immune cell types between PD and control groups. Statistical comparisons between groups were performed using the two-sided Wilcoxon rank-sum test. * *p* < 0.05; (**B**) Left panel: stacked heatmap illustrating the fraction of each immune cell type across samples. Right panel: bar plot displaying Pearson correlation coefficients between HSP90AA1 expression level and each immune-cell proportion; the adjacent color bar indicates correlation magnitude. * *p* < 0.05.

We next evaluated peripheral immune cell composition in PD blood using CIBERSORT. PD patients exhibited only a modest increase in neutrophil proportions (*p* < 0.05) and marginal changes in plasma cell frequencies compared to controls, with small overall effect sizes (Figure 4A). Notably, HSP90AA1 expression showed no significant correlation with any immune cell subtype (Figure 4B). Given that HSP90AA1 was identified as a conserved stress-responsive candidate shared between IBD and PD, yet showed no significant association with peripheral immune cell subtypes in PD blood, we hypothesized that its disease relevance might involve CNS-resident cell populations rather than circulating immune cells. As PD is fundamentally a neurodegenerative disorder with prominent glial involvement in the substantia nigra, we next examined the cellular expression patterns of HSP90AA1 in the PD substantia nigra using scRNA-seq.

### 2.4. HSP90AA1 Transcript Upregulation Is Enriched in Astrocytes of the PD Substantia Nigra

**Figure 5:**
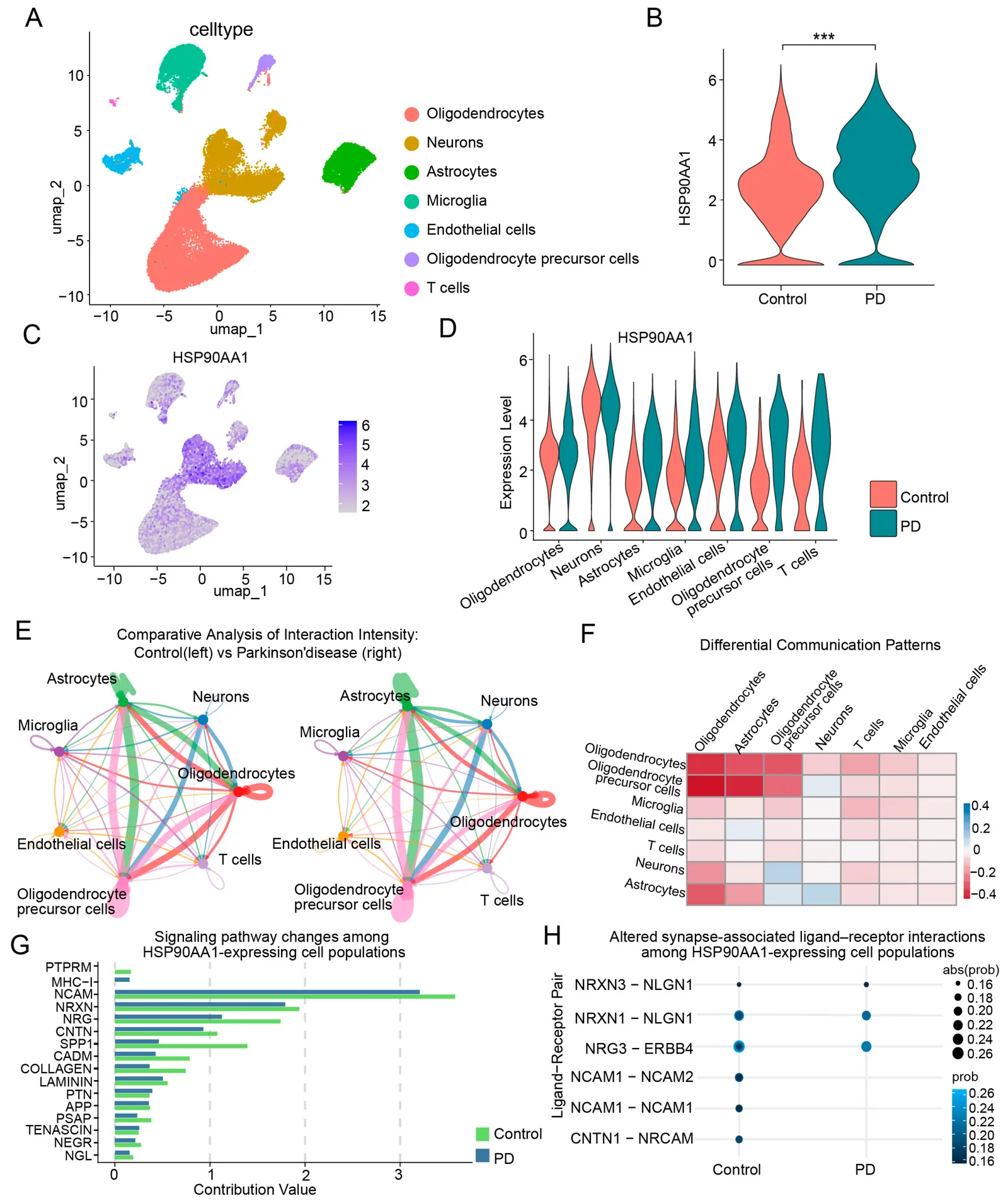
Single-cell transcriptomic characterization of HSP90AA1 expression and intercellular communication changes in the PD substantia nigra. (**A**) UMAP projection of single cells, colored by distinct cell clusters. (**B**) Comparison of HSP90AA1 expression levels between control and PD patients. Statistical comparisons between groups were performed using the two-sided Wilcoxon rank-sum test. *** *p* < 0.001. (**C**) UMAP visualization of HSP90AA1 expression (color intensity indicates expression level). (**D**) Violin plots showing HSP90AA1 expression patterns across major cell types in control and PD samples. (**E**) Comparison of cell interaction intensity in the midbrain substantia nigra between the control (**left**) and PD (**right**) groups. Node colors represent different cell types; line thickness and color indicate the strength of intercellular communication. (**F**) Heatmap showing differential cell–cell communication patterns between control and PD groups. (**G**) Signaling pathway analysis of cell–cell communication involving HSP90AA1-expressing cell populations, including astrocytes, oligodendrocytes, neurons, microglia, and T cells. (**H**) Differential ligand–receptor interactions involving HSP90AA1-expressing cell populations between control and PD groups, highlighting altered synapse-associated signaling pathways.

Following quality control (QC) and batch effect correction (Harmony, Supplementary Figure S3A), the scRNA-seq data were integrated and clustered, revealing seven major cell populations within the SNpc. These were annotated as T cells (CD3D, CD3E, TRAC, TRBC1, CD4, BCL11B, CD247, SKAP1), astrocytes (AQP4, ADGRV1, GPC5, RYR3, GFAP), microglia (C3, LRMDA, CSF1R, P2RY12, CX3CR1), oligodendrocytes (MBP, PLP1, ST18, OPALIN), oligodendrocyte precursor cells (OLIG1, PCDH15, MEGF11, VCAN), neurons (RBFOX1, RBFOX3, SYT1), endothelial cells (VWF, PECAM1, PLPP1, PTPRB, CLDN5, ABCB1, EBF1) (Figure 5A and Figure S3B).

HSP90AA1 showed detectable expression across multiple cell populations in the SNpc, including astrocytes, oligodendrocytes, microglia, neurons, and T cells (Figure 5B–D). Unlike the other five hub genes, which displayed minimal to undetectable expression in the SNpc (Supplementary Figure S4), HSP90AA1 demonstrated widespread cellular distribution and significant upregulation in PD samples, establishing it as the primary candidate for downstream cell-context analyses.

### 2.5. Cell–Cell Communication Alterations Associated with HSP90AA1-Expressing Cell Populations in PD Substantia Nigra

CellChat analysis revealed globally reduced intercellular communication intensity in PD brain tissue, with significant decreases in the number and strength of ligand-receptor interactions (Figure 5E and Figure S5A), particularly between oligodendrocytes and astrocytes (Figure 5F). Given that HSP90AA1 was broadly expressed across multiple cell types in the SNpc, we next asked whether intercellular communication involving these HSP90AA1-expressing populations was altered in PD. Among HSP90AA1-expressing populations, altered ligand–receptor communication patterns were observed. Several synapse-associated signaling pathways, including NCAM, NRXN, NRG, and CNTN, with ligand–receptor pairs such as NCAM1-NCAM2 and CNTN1-NRCAM among those showed reduced interaction probabilities in PD compared with controls (Figure 5G,H). These results suggest that altered synaptic signaling may accompany HSP90AA1 upregulation in PD.

### 2.6. TP53 Is Predicted as an Upstream Regulator of HSP90AA1 in Astrocytes

**Figure 6:**
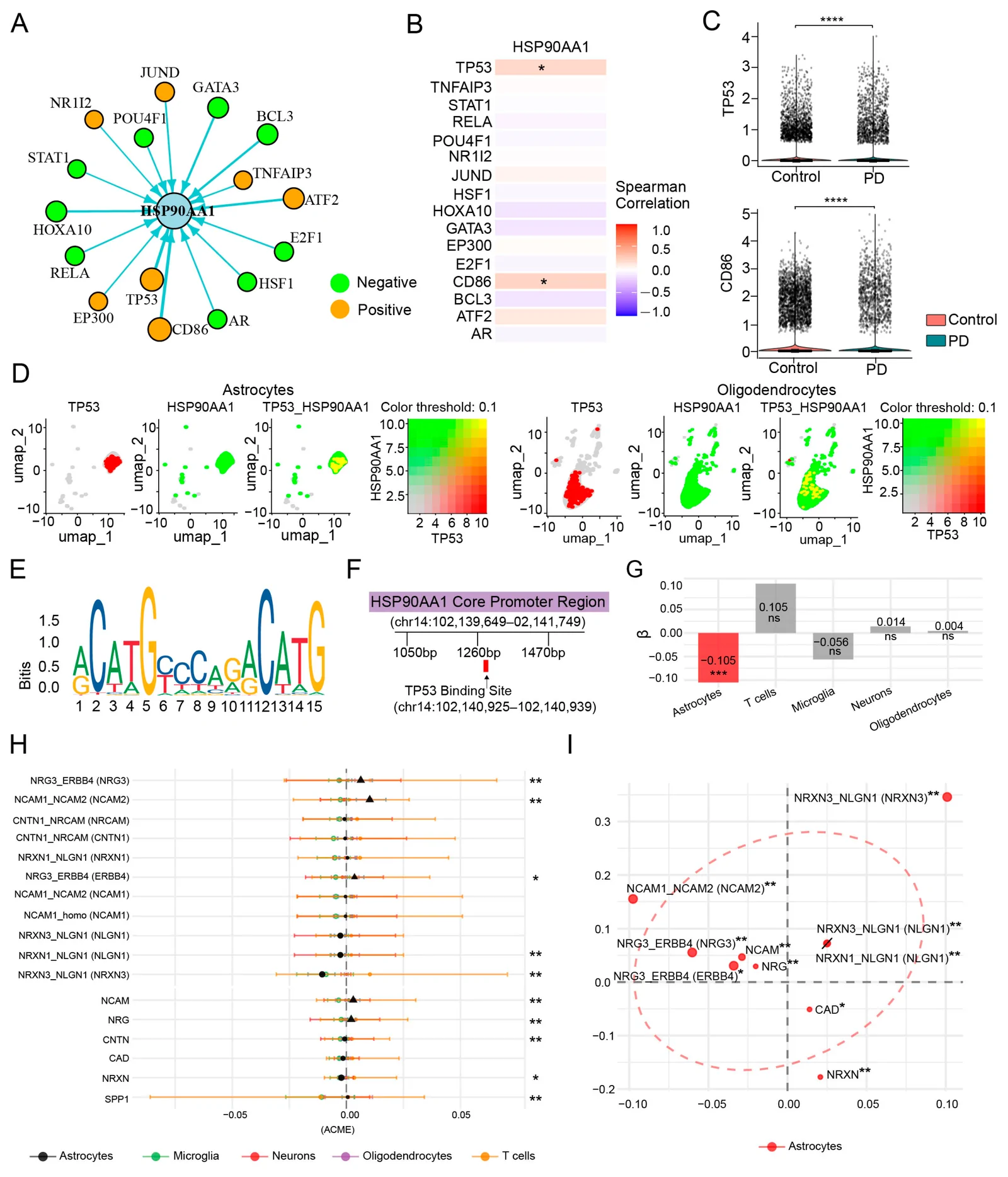
Identification of TP53 as a key transcription factor regulating HSP90AA1 and its cell-specific mediation effect. (**A**) Network diagram illustrating potential upstream transcription factors (TFs) that regulate HSP90AA1. **(B**) Heatmap showing the correlation between HSP90AA1 and candidate TFs, with a focus on TP53. Correlations were assessed using Spearman’s rank correlation coefficient. * *p* < 0.05. (**C**) Comparison of the core TF (TP53) and CD86 expression levels between control and PD groups in single-cell datasets. Statistical comparisons between groups were performed using the two-sided Wilcoxon rank-sum test. **** *p* < 0.0001. (**D**) Co-localization of HSP90AA1 and TP53 in HSP90AA1-high cells (astrocytes and oligodendrocytes). (**E**) JASPAR prediction of TP53 binding sequences. Each letter represents a nucleotide base (A, C, G, T). (**F**) Schematic diagram indicating the location of TP53 binding sites in the HSP90AA1 promoter region. (**G**) Cell type-specific regulation of HSP90AA1 by TP53 especially in astrocytes. Simple linear regression was performed separately within each cell population. Bar height represents the regression coefficient (β) of TP53; statistical significance of the regression coefficient was derived from the *t*-test implemented in linear regression. *** *p* < 0.001. ns, not significant. (**H**) Comprehensive mediation effect dot plot: TP53 regulates synapse-related molecules via HSP90AA1. Causal mediation analysis with 1000 bootstrap resamples was used to calculate the average causal mediation effect (ACME). Colored markers represent different cell types. Symbols denote estimated ACME values, and horizontal lines indicate bootstrap confidence intervals. Asterisks shown on the right indicate statistically significant mediation effects detected in astrocytes. Partial overlap of data points exists and does not affect scientific interpretation. (**I**) Dot plot showing HSP90AA1 mediation strength and its downstream effects. The red dashed circle highlights astrocyte-related gene interaction pairs. Asterisks adjacent to gene labels mark gene pairs with statistically significant mediation effects in astrocytes. Mediation effects were quantified by bootstrap-based causal mediation analysis. Significance notation: * *p* < 0.05, ** *p* < 0.01, *** *p* < 0.001, **** *p* < 0.0001.

To further define the cellular context potentially associated with HSP90AA1 regulation, we integrated transcription factor prediction with cell-type-specific co-expression and colocalization analyses. Potential TFs were identified via the TRRUST database (Figure 6A). Correlation analysis identified significant correlation of HSP90AA1 with TP53 and *CD86* (*p* < 0.05; Figure 6B), both of which were upregulated in PD substantia nigra (Figure 6C). Because CD86 is not a transcription factor, TP53 was prioritized as the most plausible upstream regulator.

Colocalization analysis revealed overlapping expression patterns between TP53 and HSP90AA1 across glial populations. Although HSP90AA1 was detectable in several cell types, the strongest TP53–HSP90AA1 co-expression and spatial colocalization signals were observed in astrocytes (Figure 6D and Figure S5B–D). Therefore, astrocytes were selected for further investigation of the potential TP53–HSP90AA1 regulatory relationship. JASPAR predicted TP53 binding sequences (Figure 6E) and identified TP53 binding sites in the core promoter region of HSP90AA1 (Figure 6F). Notably, this predicted regulatory relationship was specific to astrocytes (Figure 6G). Mediation analysis further suggested that HSP90AA1 may mediate the association between TP53 and synapse-related ligand-receptor pairs and pathways in astrocytes (Figure 6H,I). Together, these bioinformatic predictions support a putative astrocyte-enriched TP53–HSP90AA1 axis associated with synaptic dysfunction.

### 2.7. MPTP-Induced PD Mouse Model Provides Histological Support for Astrocytic HSP90α and p53 Alterations

**Figure 7:**
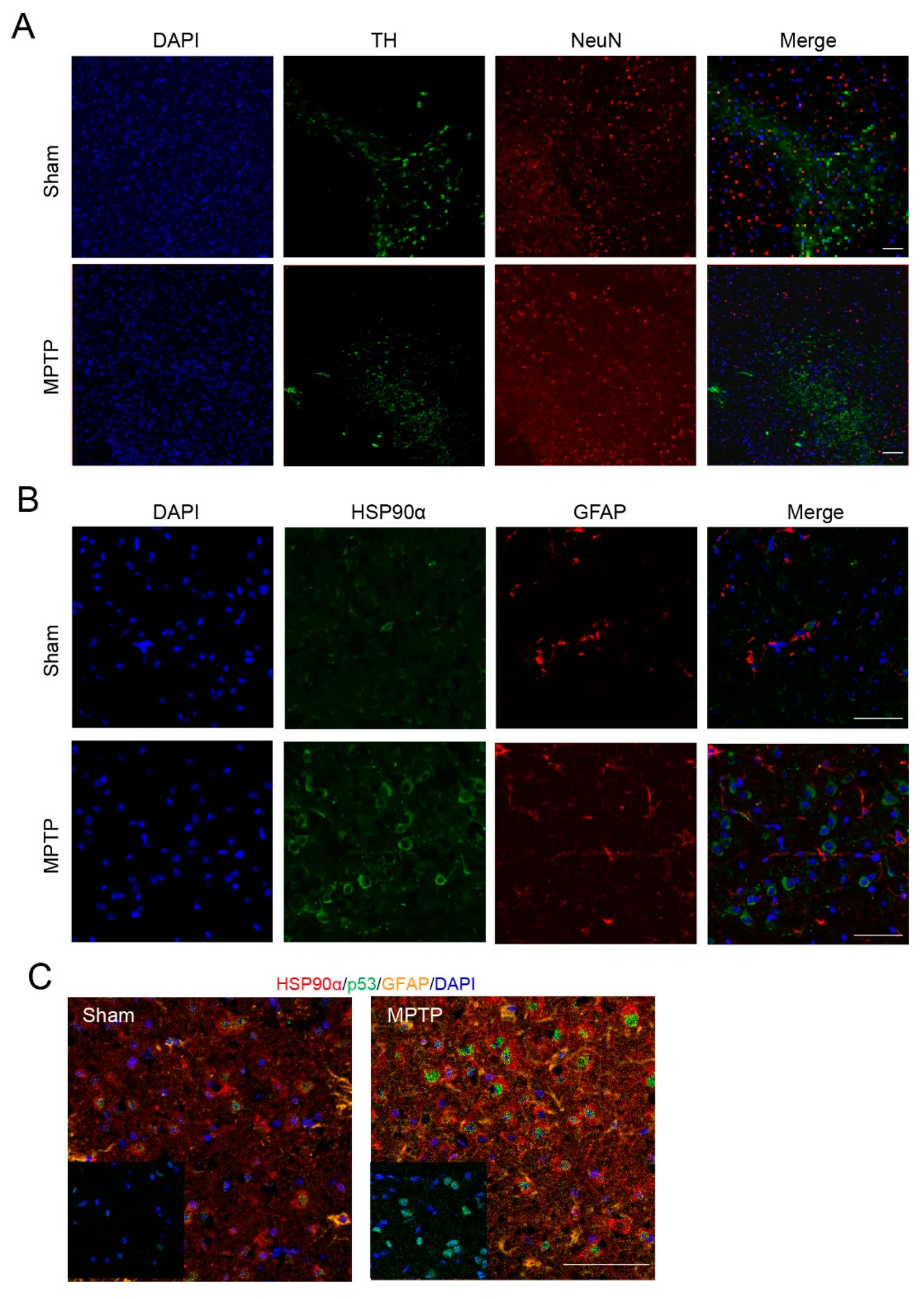
Immunofluorescence validation in MPTP-induced PD mouse model. (**A**) Representative images of TH (green) and NeuN (red) co-staining in SNpc (10× objective). (**B**) Representative images of HSP90α (green) and GFAP (red) co-staining (20× objective). (**C**) Representative TSA-based multiplex labeling of HSP90α (red), p53 (green), and GFAP (yellow) in sham and MPTP-induced PD mice. Nuclei were counterstained with DAPI (blue) (40× objective). The inset shows a magnified view of p53 (green) and DAPI (blue) merge, demonstrating increased p53-DAPI colocalization in MPTP-treated mice. Scale bar = 100 µm.

To experimentally support our multi-omics findings, we performed immunofluorescence staining on SNpc sections from MPTP-treated mice and sham controls. First, TH/NeuN co-staining revealed a reduced signal of TH^+^/NeuN^+^ dopaminergic neurons in MPTP-treated mice compared with controls (Figure 7A). Critically, HSP90α/GFAP co-staining demonstrated consistently higher HSP90α fluorescence intensity within GFAP^+^ astrocytes in MPTP-treated mice relative to controls (Figure 7B), providing histological evidence consistent with astrocytic HSP90α enrichment. We further examined p53 localization and observed increased p53 immunoreactivity, colocalized with DAPI within GFAP^+^ astrocytes in MPTP-treated mice compared with controls (Figure 7C). These in vivo observations are consistent with the predicted TP53–HSP90AA1 axis and provide experimental support for astrocytic activation in the MPTP model.

### 2.8. Proposed Model

**Figure 8:**
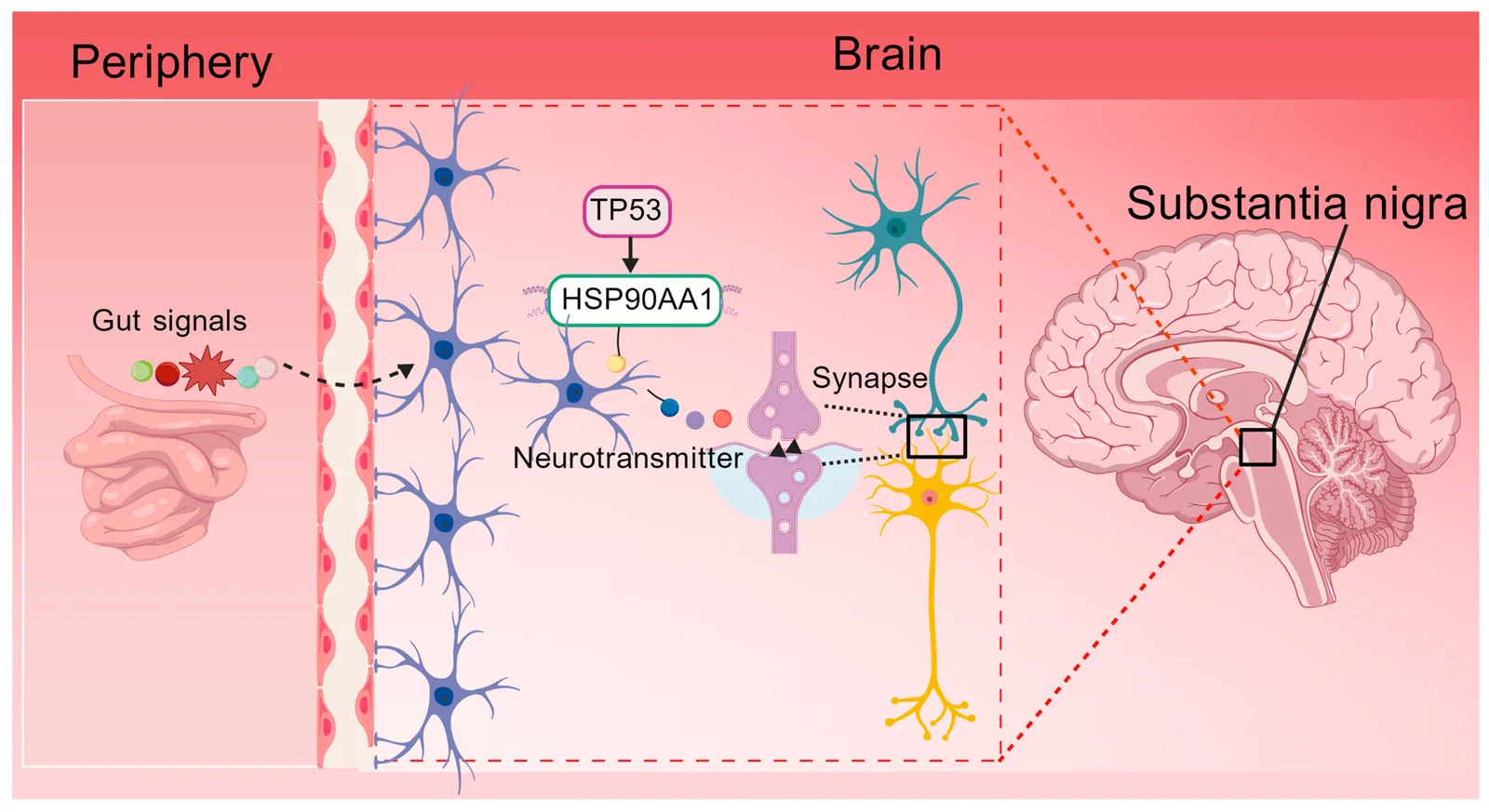
Mechanism hypothesis diagram (created with BioGDP.com). Schematic working model. Peripheral inflammatory signals may act on astrocytes in the substantia nigra. Bioinformatic analyses predict that TP53 may regulate HSP90AA1 expression in astrocytes. Elevated astrocytic HSP90AA1 is associated with altered synapse-related intercellular communication patterns, potentially contributing to synaptic dysfunction in dopaminergic neurons.

Figure 8 summarizes a working model integrating the multi-omics findings and experimental validation. HSP90AA1 was identified as a conserved stress-responsive hub shared between PD and IBD. Single-cell analysis revealed that HSP90AA1 is expressed across multiple cell populations in the PD substantia nigra. Integration of transcription factor prediction, cell-type-specific co-expression, and colocalization analyses suggested astrocytes as the major cellular context associated with the TP53–HSP90AA1 regulatory relationship. Elevated astrocytic HSP90AA1 was further associated with altered synapse-related communication patterns. Together, these findings support a testable model in which an astrocyte-associated TP53–HSP90AA1 signaling module may contribute to synaptic dysfunction in PD.

## 3. Discussion

In this study, we identified HSP90AA1 as a conserved stress-responsive molecular hub that is upregulated in both IBD and PD. Through integrated transcriptomic analysis, we further demonstrated that HSP90AA1 expression was increased in the PD substantia nigra and displayed broad expression across multiple neural and glial populations, with astrocytes showing the strongest association with the predicted TP53–HSP90AA1 regulatory pattern. Bioinformatic analysis predicted a TP53 associated regulatory component, and immunofluorescence in an MPTP-induced mouse model provided histological support for astrocytic HSP90α upregulation alongside dopaminergic neuron loss. Collectively, these findings suggest a putative astrocyte-enriched TP53–HSP90AA1 signaling module.

Our initial shared signature screening was performed using peripheral blood transcriptomic profiles from PD and IBD patients, while independent cross-cohort expression validation was conducted with post-mortem human substantia nigra brain tissue datasets. The consistent upregulation of HSP90AA1 observed across peripheral inflammatory blood and degenerative brain lesions supports its identity as a broadly conserved stress-responsive molecular hub, rather than a transient, tissue-specific expression change limited to a single compartment. HSP90AA1 encodes the inducible isoform of HSP90, a molecular chaperone that is dynamically upregulated under conditions of cellular stress or homeostatic disruption [23,24]. Its canonical function involves facilitating the proper folding, maturation, and degradation of client proteins, including α-synuclein, the major component of Lewy bodies in PD [25]. Previous studies have implicated HSP90 in neurodegenerative diseases: it colocalizes with astrocytes and microglia in Alzheimer’s disease and has been linked to synapse loss through glial-mediated engulfment of synaptic structures [26,27,28]. In PD, HSP90 has been reported as a marker of microglial activation [29]. Our findings extend these observations by suggesting that astrocytes may represent an additional cellular context in which HSP90AA1-associated stress responses occur in PD. Importantly, this prioritization was not based solely on HSP90AA1 expression abundance, but rather on the convergence of TP53 correlation, and cellular colocalization patterns.

A key finding of our study is the association between elevated HSP90AA1 and computationally predicted alterations in neuron–glia communication. CellChat analysis identified altered ligand–receptor interaction patterns involving HSP90AA1-expressing cell populations. Reduced interactions in synapse-related pathways including NCAM, NRXN, and CNTN were observed in PD, with ligand–receptor pairs such as NCAM1-NCAM2 and CNTN1-NRCAM among those affected (Figure 5G,H). These pathways contribute to synaptic adhesion, maintenance, and plasticity. Although these computational predictions cannot establish direct functional impairment, they provide a framework suggesting that astrocytic stress states accompanied by HSP90AA1 elevation may be associated with altered neuron–glia communication. This interpretation aligns with emerging concepts of astrocyte reactivity in neurodegeneration, where reactive astrocytes may lose their homeostatic supportive functions and contribute to synaptic pathology. However, these conclusions are based on computational predictions; future studies using electrophysiology or synaptic morphology assessments are warranted.

To explore the upstream regulation of HSP90AA1, we used the TRRUST database and identified TP53 as a candidate transcription factor. Correlation analysis identified a significant association between TP53 and HSP90AA1 expression (*p* < 0.05), and co-localization analysis revealed prominent overlap of TP53 and HSP90AA1 in astrocytes and oligodendrocytes (Figure 6D). Although several cell types exhibited co-expression patterns, cell-type-specific analysis showed that the TP53–HSP90AA1 regulatory correlation was strongest in astrocytes (Figure 6G), supporting their prioritization for subsequent validation. JASPAR analysis predicted TP53 binding sites in the HSP90AA1 core promoter (Figure 6E,F), and mediation analysis suggested that HSP90AA1 may bridge TP53 and synapse-related ligand–receptor pairs in astrocytes (Figure 6H,I).

TP53 is a well-established stress sensor that has been implicated in PD pathogenesis, including the regulation of α-synuclein transcription [30] and mediation of dopaminergic neuronal damage [31]. In our MPTP mouse model, we observed not only astrocytic HSP90α upregulation but also increased nuclear-associated p53 immunoreactivity within GFAP-positive astrocytes, providing preliminary in vivo evidence consistent with this predicted regulatory relationship. However, we emphasize that direct experimental evidence—such as chromatin immunoprecipitation or reporter assays—is required to confirm that TP53 transcriptionally activates HSP90AA1 in astrocytes. Therefore, the TP53–HSP90AA1 relationship identified here should be interpreted as a testable regulatory hypothesis rather than an established mechanism.

We initially identified HSP90AA1 through a cross-disease screen between PD and IBD, a classic model of chronic intestinal inflammation. Epidemiological and experimental evidence has long suggested a link between gastrointestinal inflammation and PD risk [12,32,33,34], and the gut–brain axis hypothesis proposes that peripheral pathology may influence central neurodegeneration [35,36]. Our finding that HSP90AA1 is upregulated in both IBD and PD supports the concept that conserved stress-responsive programs can be activated across tissues. Recent epigenetic research highlights inflammation-associated microRNAs as critical messengers bridging intestinal inflammation and central glial dysfunction. Miquel-Rio et al. [37] uncovered a convergent miRNA signature shared by PD, gut dysfunction and PD-related psychiatric comorbidities, where dysregulated circulating miRNAs trigger NF-κB-mediated glial inflammatory cascades similar to our TP53–HSP90AA1 stress module.

Several limitations should be acknowledged. First, the predicted transcriptional regulation requires further validation using direct approaches such as ChIP-qPCR or Tp53 siRNA knockdown experiments. Second, CellChat analysis provides hypothesis-generating evidence based on ligand–receptor expression patterns and does not directly demonstrate functional synaptic impairment. Third, the MPTP model represents an acute neurotoxic PD model and does not fully reproduce the chronic inflammatory processes proposed in the gut–brain axis hypothesis. In addition, our in vivo validation was performed exclusively in male mice, and future studies including both sexes are needed to determine whether the observed astrocytic stress response exhibits sex-dependent differences. Lastly, the relatively small sample sizes, particularly the four animals per group in the MPTP model and the three patients per group in the scRNA-seq dataset, should be acknowledged as a limitation to the generalizability of our findings.

Despite these limitations, our integrated transcriptomic analysis provides a coherent framework identifying an astrocyte-enriched TP53–HSP90AA1 stress-response module as a candidate contributor to altered synaptic communication in PD, and establishes a testable hypothesis for future mechanistic studies.

## 4. Materials and Methods

### 4.1. Data Selection

For PD, the gene expression profiling by array datasets GSE6613 and GSE22491 were downloaded from the GEO database (https://www.ncbi.nlm.nih.gov/geo/) accessed on 23 August 2024, including 60 PD samples and 31 healthy control samples. For IBD, dataset GSE3365 was obtained from GEO, which contains 85 IBD samples and 42 healthy control samples accessed on 4 September 2024. The two PD datasets were merged, normalized, and batch effects were removed using the ComBat package. PD datasets GSE7621 (16 PD patients and 9 controls) and GSE20163 (8 PD patients and 9 controls) were used for validation accessed on 27 September 2024. Single-cell RNA sequencing data from PD patients and healthy controls were obtained from GSE243639 for downstream analysis accessed on 19 October 2024. Details are shown in Table 1.

### 4.2. Construction of a Weighted Gene Co−Expression Network

Weighted gene co-expression network analysis was used to build unsigned co-expression networks. Genes with variance lower than the 25th quantile were discarded. Candidate soft threshold powers ranging from 1 to 20 were tested. For the PD dataset, a soft threshold power of 5 was selected based on scale-free topology criteria (R^2^ > 0.90). For the IBD dataset, a soft threshold power of 8 was selected according to the same criterion. Gene modules were partitioned by dynamic tree cutting with min Module Size = 100 and merge Cut Height = 0.25. Module eigengenes were correlated with PD/IBD phenotypic traits using Pearson correlation. Student asymptotic *p*-values were calculated for module–trait correlations, and nominal *p* values were reported due to the limited number of module–trait comparisons.

### 4.3. Functional Enrichment Analysis of Shared Co-Expression Genes

GO annotation including cellular component (CC), biological process (BP), molecular function (MF), and KEGG pathway enrichment analysis were applied using R software (v4.2.2) to further clarify the potential functional annotation and pathway enrichment. The Benjamini–Hochberg method was applied for multiple testing correction. Terms with adjusted *p* value < 0.05 were considered statistically significant.

### 4.4. PPI Network Construction and Hub Genes Screening and Validation

The STRING online Search Tool for the Retrieval of Interacting Genes database tool (STRING-DB) (http://string-db.org/, accessed on 19 October 2024) was used to analyze PPI information with the threshold of the combined score > 0.4. PPI network was visualized using the CytoHubba plugin of Cytoscape (v3.9.1). Four algorithms, including MNC, MCC, EPC, and Degree, were used to identify candidate hub genes. The common genes obtained from the top 10 identified genes by the four algorithms were considered as the shared hub genes in our study, and demonstrated using a Venn diagram. PD validation dataset GSE7621, GSE20163 were used to verify the expression level of hub genes in brain tissue cohorts.

### 4.5. Evaluation of Immune Cell Infiltration

CIBERSORT is a versatile computational method widely used to quantify cell fractions from bulk tissue gene expression profiles (GEPs) [38]. We used CIBERSORT to analyze the proportion of 22 immune cells in PD datasets. The expressed levels of the different immune cells were visualized via the “ggplot2” package of R software.

### 4.6. Single-Cell Sequencing Analysis

For single-cell sequencing analysis, raw data for GSE243639 were downloaded from the GEO database and processed using Seurat (v5.0.3) in R (v4.2.2). Three PD patients and three healthy controls were selected for balanced comparative analysis.

For cell quality control filtering: only cells with 200–4000 uniquely detected genes, total UMI counts below 30,000, and mitochondrial gene expression proportion less than 20% were retained for downstream analysis. Counts were log-normalized with a scale factor of 10,000, and the top 2000 highly variable genes were identified using the variance-stabilizing transformation (vst) method. Data were scaled, and principal component analysis (PCA) was performed. Batch effects across samples were corrected using Harmony (v4) with default parameters. The top 20 harmony-aligned dimensions were used for UMAP dimensionality reduction and graph-based clustering at resolution 0.1. Marker genes for each cluster were identified using the FindAllMarkers function with the Wilcoxon rank-sum test and Bonferroni correction. Genes with |avg_log2FC| > 0.3 and adjusted *p* < 0.05 were defined as marker genes. In total, 7 cell clusters were identified and visualized using the UMAP algorithm. Cell populations were annotated manually based on established marker genes. After clustering and cell annotation, the expression distribution and cellular localization of HSP90AA1 were evaluated across all annotated substantia nigra cell populations. Differences in HSP90AA1 expression between PD and control groups were analyzed using the Wilcoxon rank-sum test.

### 4.7. Cell–Cell Communication Analysis

For the inference and analysis of cell–cell communication, we used CellChat (1.6.1), a public repository of ligands, receptors, cofactors, and their interactions [39]. CellChat was applied to identify potential differences in intercellular communication between PD and control samples. As HSP90AA1 showed detectable expression across multiple cell types in the PD substantia nigra, we first evaluated global HSP90AA1 expression across all cell subtypes via violin plots. We then selected five cell types with prominent HSP90AA1 expression (astrocytes, oligodendrocytes, neurons, microglia, and T cells) for targeted comparison of cell–cell communication patterns between PD and control groups. Ligand-receptor pairs with interaction probability > 0.15 were retained for visualization.

### 4.8. TFs-Hub Gene Regulatory Relationship Validation

The TRRUST database was utilized to predict TFs. Potential upstream TFs of HSP90AA1 were predicted using TRRUST and further assessed by Spearman’s correlation analysis (adjusted *p* < 0.05). Cell-type co-expression in astrocytes was examined using scRNA-seq. TF binding sequences were predicted based on the JASPAR database. The positional association between TF binding sites and the HSP90AA1 promoter region was investigated via the UCSC Genome Browser. These analyses were used to explore potential TF–HSP90AA1 regulatory relationships.

### 4.9. MPTP-Induced PD Mouse Model

Male mice were selected because female C57BL/6J mice exhibit substantially higher resistance to MPTP-induced dopaminergic toxicity, attributable to estrogen-mediated neuroprotective effects on nigrostriatal dopaminergic neurons. Mice (20–25 g, *n* = 4 per group) were housed in a specific-pathogen-free (SPF) animal facility under standard laboratory conditions (22–24 °C, 12 h light/dark cycle, 50–60% humidity) with free access to standard rodent chow and sterile drinking water. To establish an acute PD model, mice received intraperitoneal MPTP injections with a total dose of 80 mg/kg administered as four separate injections at 2 h intervals on a single day. All animals were deeply anesthetized and transcardially perfused on day 7 after MPTP treatment, and brain tissues were harvested for histological analysis. Serial substantia nigra sections were prepared for TH immunohistochemistry and GFAP/HSP90α/p53 immunofluorescence staining. Immunofluorescence analyses were performed primarily for spatial localization and qualitative validation of computational findings. All animal experimental procedures were approved by the Institutional Animal Care and Use Committee of Beijing Tiantan Hospital (202203004) and strictly performed in accordance with the ARRIVE 2.0 guidelines. All tissue sectioning, staining procedures, and image acquisition were conducted by investigators blinded to group assignments to eliminate observation bias.

### 4.10. Immunofluorescence

Sections were incubated with anti-TH (1:400, Abcam, ab137869, Cambridge, UK) and anti-NeuN (1:500, Abcam, ab104224) for TH/NeuN co-staining, or anti-HSP90α (1:100, Abmart, PA1558, Shanghai, China) and anti-GFAP (1:500, Abcam, ab7260) for HSP90α/GFAP co-staining. After washing, sections were incubated with species-appropriate Alexa Fluor-conjugated secondary antibodies: donkey anti-rabbit Alexa Fluor 488 and donkey anti-mouse Alexa Fluor 594 (1: 400, Invitrogen, Waltham, MA, USA). Nuclei were counterstained with DAPI.

To achieve multi-target co-localization detection, tyramide signal amplification (TSA) was applied with a commercial TSA kit: the NEON^®^ DendronFluor TSA 7-Color Multiplex Fluorescence Kit (Cat. No. QZDFT7C100, Bodubio Biotechnology Co., Ltd., Beijing, China). Sections were incubated with a primary antibody cocktail containing anti-HSP90α (1:100), anti-p53 (1:50, Abmart, TA0879), and anti-GFAP (1:500), followed by HRP-conjugated secondary antibody and sequential fluorophore-conjugated tyramide deposition. Nuclei were counterstained with DAPI.

All fluorescence images were obtained from independent biological replicates. Given the exploratory validation design, immunofluorescence results were interpreted as qualitative morphological evidence supporting transcriptomic and single-cell findings.

### 4.11. Statistical Analysis

Statistical analysis was carried out using R software and various bioinformatics packages and algorithms were utilized. *p* < 0.05 was considered to indicate statistical significance.

## 5. Conclusions

In this study, HSP90AA1 was identified as a conserved stress-responsive hub that is upregulated in PD substantia nigra. Single-cell transcriptomic analysis demonstrated that HSP90AA1 is expressed across multiple substantia nigra cell populations, while astrocytes showed the strongest association with TP53-related regulatory patterns and were therefore selected for experimental validation. Integrated transcriptomic analyses and MPTP mouse model validation suggest that astrocytic HSP90AA1 upregulation is associated with altered synaptic signaling, potentially involving a predicted TP53-mediated transcriptional regulation. These findings highlight a candidate astrocyte-enriched TP53–HSP90AA1 stress-response module that may contribute to altered synaptic communication in PD and provide a testable framework for future mechanistic investigation.

## Figures and Tables

**Table 1:** Summary of GEO datasets used in this study.

GEO Accession	Platform Information	Disease	Disease Group	Control Group	Species
GSE6613	GPL96	PD	50	23	Homo sapiens whole blood
GSE22491	GPL6480	PD	10	8	Homo sapiens peripheral blood
GSE3365	GPL96	IBD	85	42	Homo sapiens peripheral blood
GSE7621	GPL570	PD	16	9	Homo sapiens substantia nigra
GSE20163	GPL96	PD	8	9	Homo sapiens substantia nigra
GSE243639	GPL24676	PD	15	14	Homo sapiens SNpc

substantia nigra pars compacta: SNpc.

## Data Availability

The original contributions presented in this study are included in the article/Supplementary Material. Further inquiries can be directed to the corresponding authors.

## Supplementary Materials

The following supporting information can be downloaded at: https://www.mdpi.com/article/10.3390/ijms27167140/s1.
