# Supplementary material for: Astrocytic HSP90AA1 Upregulation and Altered Synaptic Signaling in Parkinson’s Disease: Transcriptomic Screening and In Vivo Validation
Source: Int J Mol Sci. 2026 Aug 9;27(16):7140. doi: 10.3390/ijms27167140 (PMC13513560; doi:10.3390/ijms27167140)
Supplement: Supplementary file 1 [file ijms-27-07140-s001.zip › ijms-4411744-supplementary.pdf]

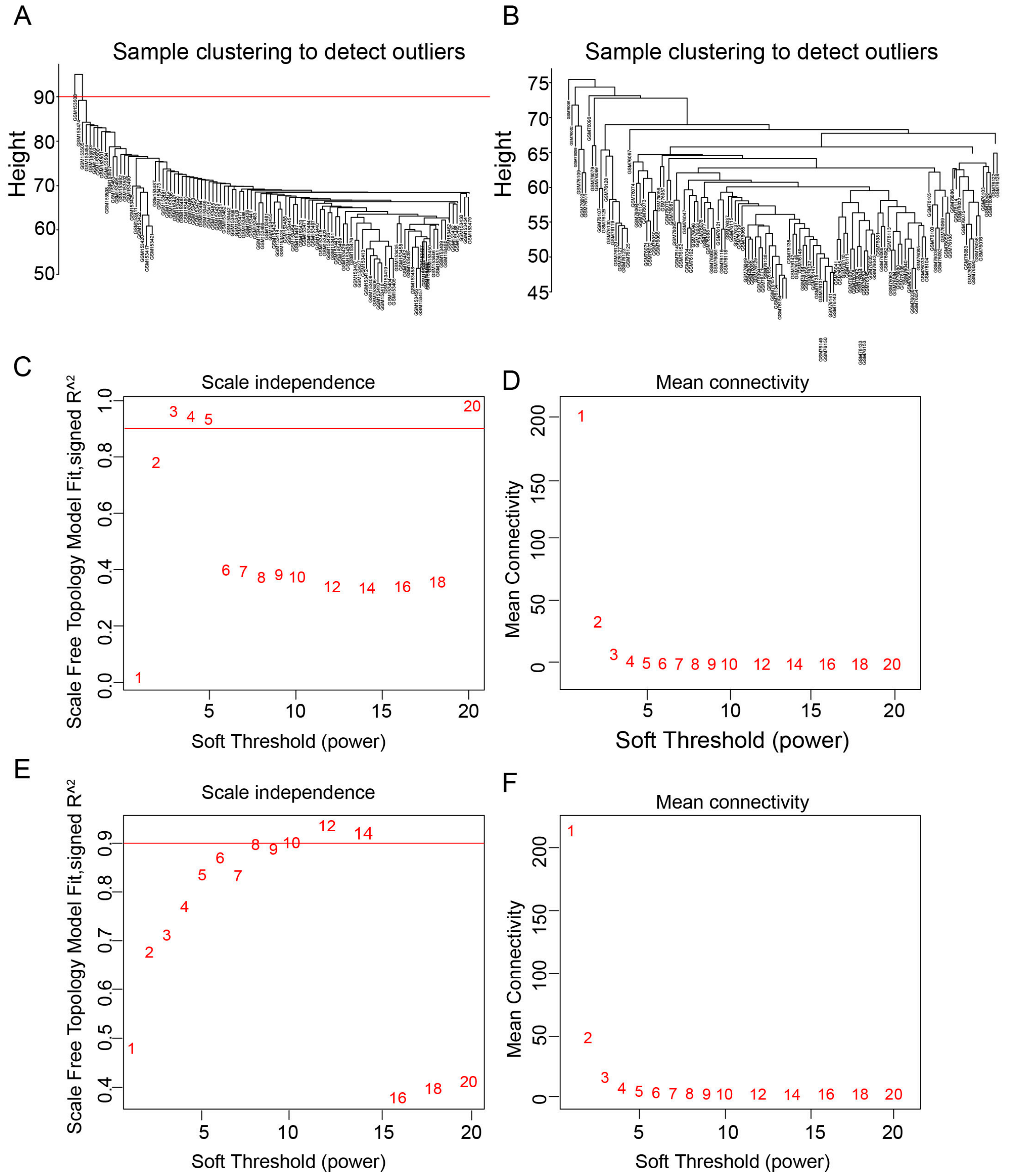

Supplementary Figure S1: Sample clustering and soft threshold selection for co-expression network construction.

(A, B) Hierarchical clustering dendrograms of PD (A) and IBD (B) patient samples based on gene expression profiles. Each branch represents one sample; one outlier sample was identified in the PD dataset. (C – F) Scaleless index and average connectivity of individual soft thresholds for PD (C, D) and IBD (E, F).

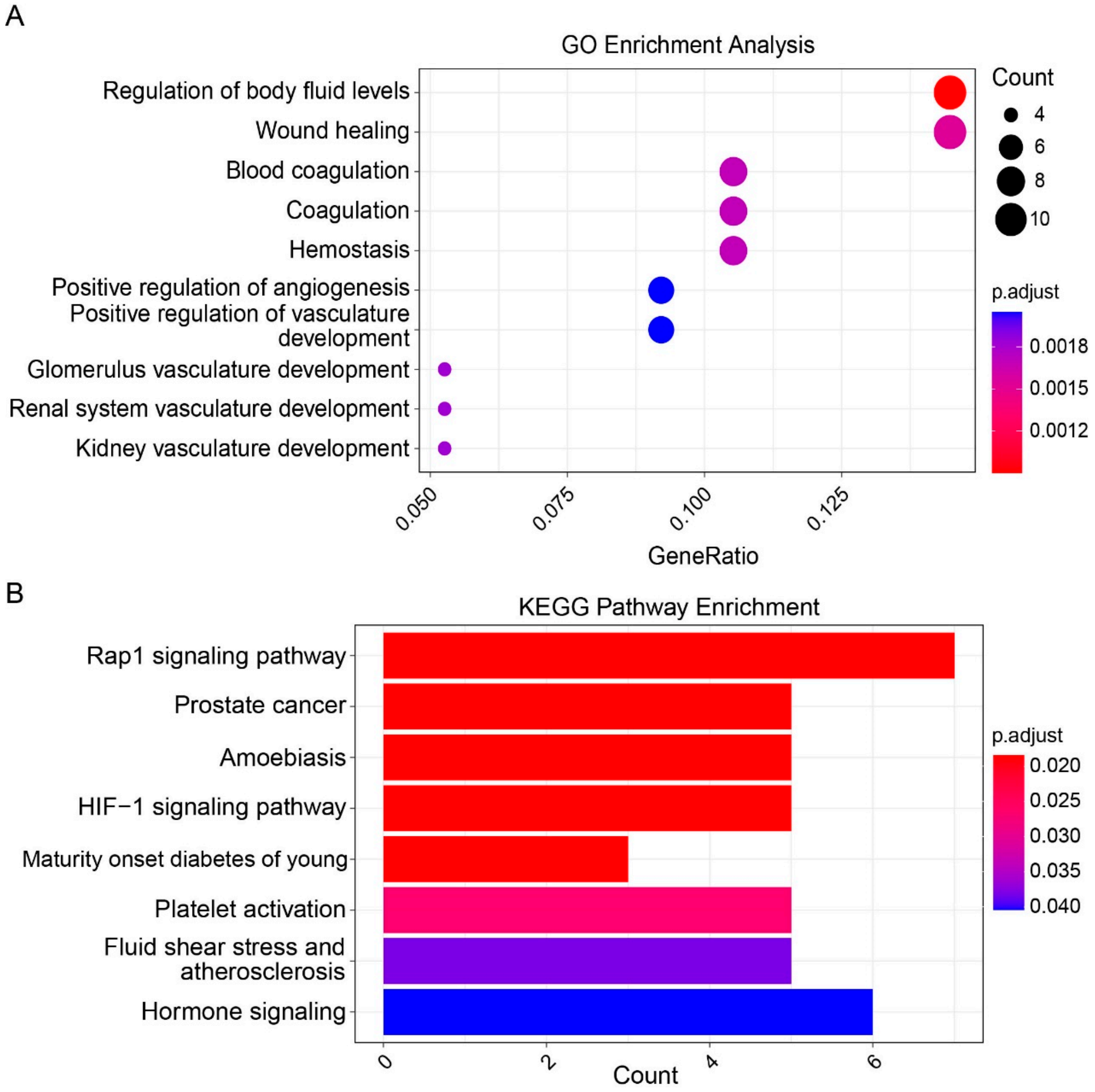

Supplementary Figure S2: Functional enrichment analysis of common genes.

(A) Gene Ontology (GO) analyses for the intersect genes of WGCNA hub genes. (B) KEGG analysis of intersect genes.

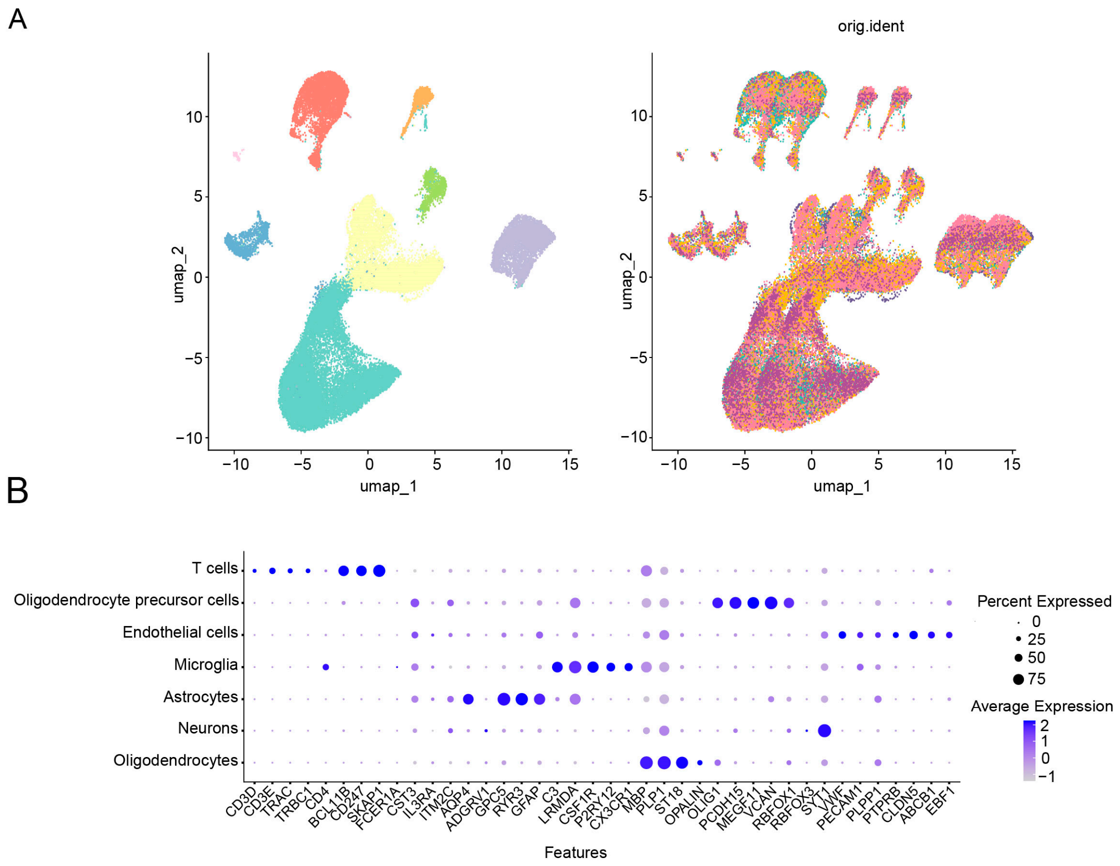

Supplementary Figure S3: Batch effect correction and cell type annotation for single-cell RNA sequencing (scRNA-seq) data.

(A) UMAP visualizations of scRNA-seq cells before (left) and after (right) batch effect correction. (B) Dot plot for cell population annotation.

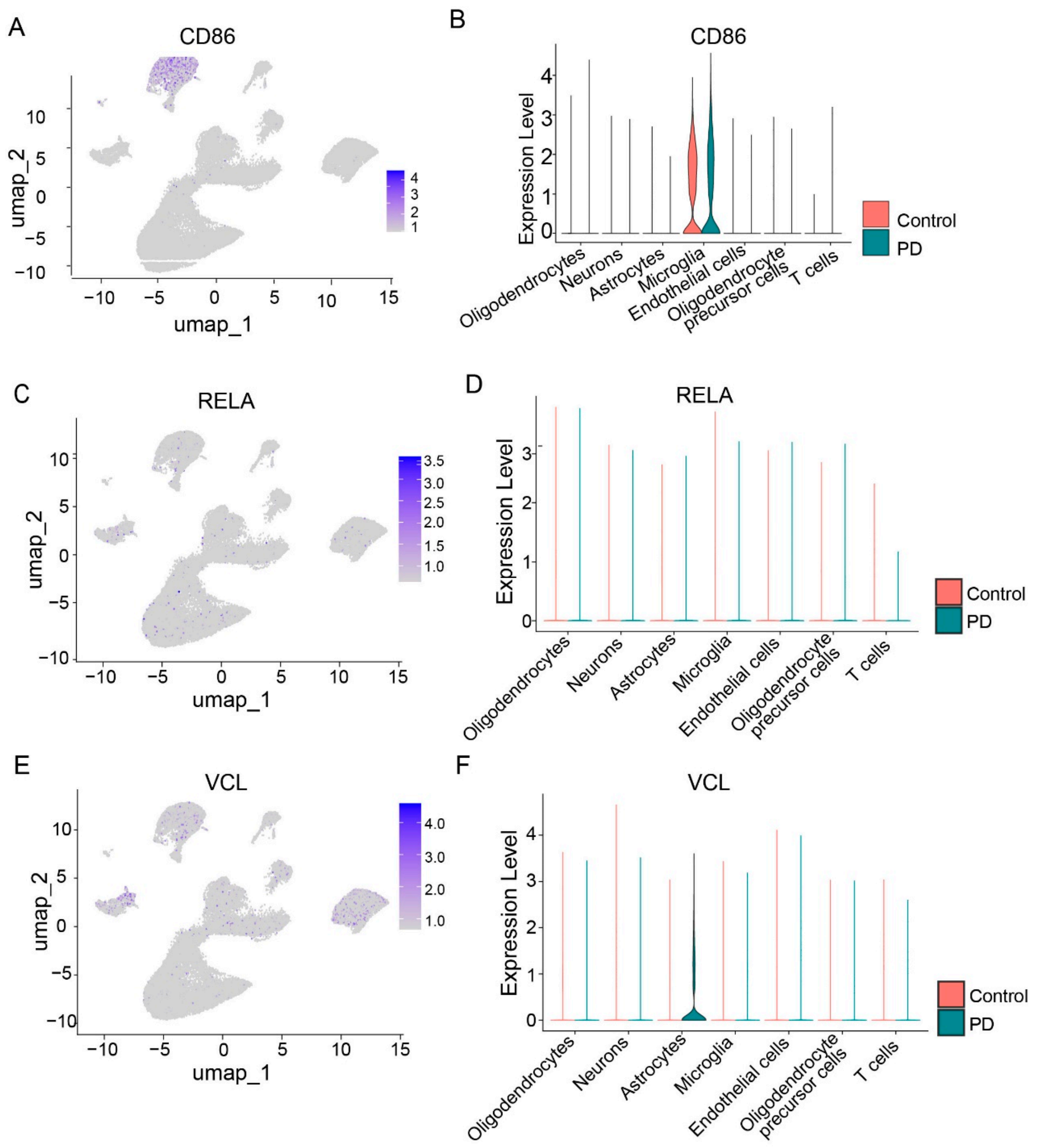

Supplementary Figure S4: Single-cell expression patterns of CD86, RELA, and VCL in PD.

(A, C, E) UMAP visualizations showing the expression of CD86(A), RELA (C), and VCL (E) across different cell types. (B, D, F) Comparison of CD86 (B), RELA (D), and VCL (F) expression levels across cell types between control and PD patients.

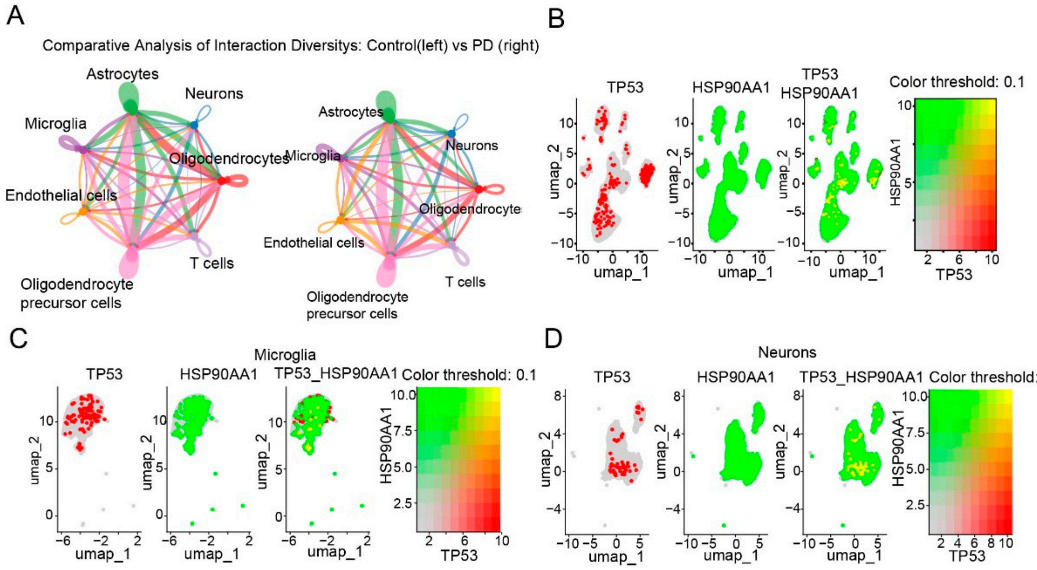

Supplementary Figure S5: Global intercellular communication and TP53-HSP90AA1 co-localization analysis.

(A) Comparative Analysis of Interaction Diversitys: Control(left) vs PD (right). (B) Co-localization of TP53 and HSP90AA1 in the total cell population. (C, D) Cell type-specific co-localization of TP53 and HSP90AA1: in microglia (C) and neurons (D).
